# Structure and diversity of phyllostomid bat assemblages on riparian corridors in a human-dominated tropical landscape

**DOI:** 10.1002/ece3.1375

**Published:** 2015-01-28

**Authors:** Erika de la Peña-Cuéllar, Julieta Benítez-Malvido, Luis Daniel Avila-Cabadilla, Miguel Martínez-Ramos, Alejandro Estrada

**Affiliations:** 1 Centro de Investigaciones en Ecosistemas, Universidad Nacional Autónoma de MéxicoMorelia, Michoacán, México; 2 Escuela Nacional de Estudios Superiores, Universidad Nacional Autónoma de MéxicoMorelia, Michoacán, México; 3 Estacion de Biología Tropical “Los Tuxtlas”, Instituto de Biología, Universidad Nacional Autónoma de MéxicoMorelia, Michoacán, México

**Keywords:** Agricultural matrix, corridors, diversity, frugivores, indicator taxa

## Abstract

Tropical forests around the world have been lost, mainly because of agricultural activities. Linear elements like riparian vegetation in fragmented tropical landscapes help maintain the native flora and fauna. Information about the role of riparian corridors as a reservoir of bat species, however, is scanty. We assessed the value of riparian corridors on the conservation of phyllostomid bat assemblage in an agricultural landscape of southern Mexico. For 2 years (2011–2013), mist-netting at ground level was carried out twice during the dry season (December to May) and twice during the wet season (June to November) in different habitats: (1) riparian corridors in mature forest, (2) riparian corridors in pasture, (3) continuous forest away from riparian vegetation, and (4) open pastures. Each habitat was replicated three times. To determine the influence of vegetation structure on bat assemblages, all trees (≥10 cm dbh) were sampled in all habitats. Overall, 1752 individuals belonging to 28 species of Phyllostomidae were captured with Sternodermatinae being the most rich and abundant subfamily. Riparian corridors in mature forest and pastures had the greatest species richness and shared 65% of all species. Open pastures had the lowest richness and abundance of bats with no Phyllostominae species recorded. Six of the 18 species recorded could be considered as habitat indicators. There was a positive relationship between bat species composition and tree basal area. Our findings suggest that contrary to our expectations, bats with generalist habits and naturally abundant could be useful detector taxa of habitat modification, rather than bats strongly associated with undisturbed forest. Also in human-dominated landscapes, the maintenance of habitat elements such as large trees in riparian corridors can serve as reservoirs for bat species, especially for those that are strongly associated with undisturbed forest.

## Introduction

Habitat loss and fragmentation are considered the most serious threats to biodiversity and the main cause of the current extinction crisis (Laurance and Bierregaard 1997). In tropical regions, many biodiversity hot spots have been converted to agricultural lands (Myers et al. 2000; Achard et al. 2002). In many of these landscapes, the presence of vegetation along streams is a common feature and is relatively resistant to agricultural practices and small-scale land use changes (Lundy and Montgomery 2010). Riparian habitats provide some of the most diverse and complex terrestrial habitats (Naiman et al. 1993). Especially, in fragmented landscapes, riparian vegetation contrasts with adjacent grassland areas as it provides the following landscape elements for the native biota: habitat for many species, corridors for flying and terrestrial animals, connectivity between forest fragments and for fostering network dispersion (Naiman et al. 2000; Estrada and Coates-Estrada 2001). In particular, bird species composition and density differ considerably between riparian vegetation and the surrounding agricultural matrix (Warkentin et al. 1995; Seaman and Schulze 2010). Similarly, bats use riparian vegetation as flyways during foraging activities, reducing the distance that they need to travel from and to their refuges (Daniel et al. 2008).

Intensification of agricultural practices in the tropics is likely to threaten the persistence of some bat species; nonetheless, the presence of landscape elements like live fences, isolated trees, and riparian vegetation disrupts the homogeneity of pastures and has been shown to be important in the maintenance of bat diversity (Harvey et al. 2006; Griscom et al. 2007; Medina et al. 2007). Riparian habitats provide flyways and foraging areas for bats, serving as stepping stones to isolated patches of primary vegetation. In addition, riparian zones offer important sources of water and food for bats (Estrada and Coates-Estrada 2001; Galindo-González and Sosa 2003).

Bats are widely studied because they play a crucial role in the ecosystem functioning as pollinators, seed dispersal agents and controllers of invertebrate and small vertebrate populations (Muscarella and Fleming 2007; Kalka et al. 2008; Kunz et al. 2011). Particularly in the Neotropics, bats are considered an important component of mammal biodiversity accounting for over 50% of the species (Medelln 1994). The response of bats to habitat loss in the Neotropics is ambiguous, compared to the Stenodermatinae (frugivore bats), and the Phyllostominae bats are very sensitive to disturbance and tend to decrease in degraded and fragmented habitats because of their limited range sizes, specialized resource needs (food and roosting), and because of their avoidance of open pastures (Kalko et al. 1999; Medellín et al. 2000; Schulze et al. 2000; Castro-Luna et al. 2007a; de la Peña-Cuéllar et al. 2012; García-Morales et al. 2013). We selected the Phyllostomidae family because it is the most species rich and functionally diverse bat family in the Neotropics. Furthermore, because of the broad spectrum of biological interactions in which they are involved, phyllostomids have been recognized as useful indicators of habitat quality (Fenton et al. 1992; Medellín et al. 2000; Jones et al. 2009).

In human-impacted landscapes, information about the importance of riparian vegetation in maintaining bat species diversity is crucial for understanding bat behavioral and ecological flexibility. In this framework, we recorded the richness and abundance of phyllostomid bats present in different habitat types within an agricultural matrix to determine the following: (1) bat assemblages attributes (i.e., species composition and species density) in riparian and nonriparian habitats; (2) the extent to which the structural complexity of the vegetation explains bat species composition; and (3) the distribution of species, genera and subfamilies in the different habitats in order to identify indicator taxa.

## Methods

### Study area and sampling sites

The study was conducted in the tropical rain forest region of Lacandona, Chiapas, Mexico. The original vegetation consists of semideciduous and lowland tropical rain forests. Mean annual temperature is 24°C, and average annual rainfall is 3000 mm with June to October as the wettest months (551 mm per month) and February to April as the driest months (<100 mm per month) (Breugel et al. 2006; CFE 2006). Deforestation of the region began in the 1970s, resulting in the reduction of closed forest from 95% in 1976 to 56% in 1996 (de Jong et al. 2000); only 36% of closed forest remains today (Carabias et al. 2008). The main practices of the region consist of grazing pastures, maize and other crops, and patches of secondary and old-growth forests (de Jong et al. 2000).

**Figure 1:**
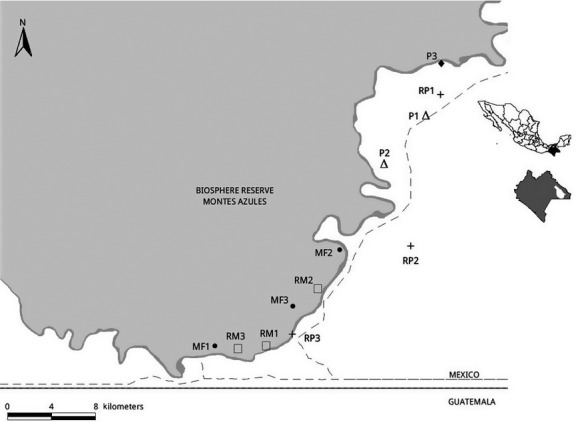
Study area and bat sampling sites at the Lacandona forest, Chiapas, Mexico.

We sampled four different habitats: (1) riparian sites within mature continuous forest (RM); (2) riparian sites in open pastures (RP); (3) mature continuous forest away from riparian vegetation (MF); and (4) open pastures (P). Each habitat type was replicated three times, and sites were at least 1.5 km away from each other. Streams were all permanent (although with variable amounts of running water throughout the year); stream width varied from two to eight meters. Study sites in pastures were located in the fragmented landscape of the Marqués de Comillas municipality, on the south side of the Lacantún River. Mature continuous forest sites were located in the 330,000 ha Montes Azules Biosphere Reserve (MABR) on the north side of the river (16°04′N–90°45′ W; Fig.1) (INE 2000).

### Bat and vegetation sampling

Bat sampling was performed twice during the dry season (December to May) and two times during the wet season (June to November) for three consecutive years (2011, 2012, and 2013). Eighteen nights were sampled at RM, RP, and P habitats, and sixteen nights were sampled at the MF habitat. Bats were surveyed at all sites using the same standardized method. Five nets (12 m long × 2.6 m high) were set at ground level and were opened at dusk for four consecutive hours, which corresponds to the peak foraging time for most phyllostomid species (La Val 1970). The nets were arranged following three configurations located roughly 50 m apart: (1) one individual net and two pairs of nets were placed in the “L” position (two nets connected perpendicularly); (2) in the riparian habitats nets were located parallel and diagonally across the stream depending on site characteristics, in the MF habitat nets were placed in natural corridors that represented flyways for bats; and 3) in P, nets were located in open spaces devoid of vegetation. Bat sampling avoids nights with a full moon or heavy rain (Morrison 1978). Captured individuals were temporarily stored in cloth bags and identified to species following Medellín et al. (2011). We used Koopman's classification (1993) for bat families, subfamilies and genera. For species and feeding guilds, we followed Timm and La Val (1998) classification (i.e., aerial insectivores, carnivores, gleaning insectivores, frugivores, nectarivores, and sanguivores).

Tree sampling was carried out once at each habitat to determine the influence of vegetation structure on bat assemblages; we recorded all trees ≥10 cm dbh within a 0.1 ha (20 × 50 m) plot (Gentry 1982). Plots were located along streams in RM and RP habitats and randomly located in MF and P habitats. We considered the following vegetation attributes: number of individuals (NI), number of species (NS), total basal area (BA), and height (H).

### Statistical analyses

#### Bat sampling completeness

We assessed the completeness of the bat survey by calculating the percentage of estimated species richness that was effectively covered by our samples. To ensure a good representation of bat richness, species richness was estimated by computing the average of the following indices: ICE, Chao2 and Bootstrap (Colwell et al. 2004). Ninety percent of completeness was considered to be a satisfactory level of sampling efficiency (Moreno and Halffter 2001).

We used a Mantel test with 999 permutations to determine whether bat assemblages closer together were more similar than those farther apart (Mantel 1967). We computed the correlation between the matrix representing the Euclidean distance among sites (represented in UTM units) and the matrix of Bray–Curtis indices representing ecological distances. These analyses were performed in R (R Development Core Team 2009) with the vegan package (Oksanen et al. 2011).

#### Bat assemblages

We built rank-abundance (dominance-diversity) plots for each habitat; these graphs have been suggested as an alternative to diversity indexes when comparing communities in different habitats (Feinsinger 2001). Individual-based rarefaction curves were constructed to compare species richness among habitats (EstimateS software, version 7.5, Copyright R. K. Colwell: http://viceroy.eeb.uconn.edu/estimates). The 95% confidence intervals of the moment-based estimator for species richness (sobs Mao Tau) were used to determine significant differences among habitats (Colwell et al. 2004).

To evaluate dissimilarity patterns among phyllostomid assemblages we used nonmetric multidimensional scaling (NMDS) based on Bray–Curtis similarity. The NMDS is one of the most appropriate ordination methods in community ecology (McCune and Grace 2002) as it can properly handle nonlinear species responses (Oksanen 2010), high beta-diversity and data not adjusted to a particular underlying model (i.e., multivariate normality), which are common in community dataset (McCune and Grace 2002). We used the stress value to evaluate the ordination. Low stress values indicate that the distances between objects in space ordination are similar to the distances between objects in the original space, defined by n-dimensions (in this case, the species considered in the matrix). The lower the stress value, the more reliable the results achieved by the ordination.

#### Phyllostomid bats as indicator taxa

We evaluated if phyllostomid taxa (subfamily, genus and species) were associated with particular habitats, and therefore considered as indicators of such habitats. Indicator taxa are characteristic of a particular habitat whereas detector taxa exhibit different degrees of preferences for different habitat types and consequently are useful in indicating habitat change. For this purpose, we performed the “indicator value analysis” (Dufrene and Legendre 1997). This method assigns an indicator value (IV) to each taxon, in each habitat, based on the taxon's relative frequency of occurrence (fidelity) and relative abundance (specificity). We then selected the maximum IV (IV_max_) for each taxon and identified the corresponding habitat. The IV_max_ statistical significance was evaluated through a Monte Carlo test based on 1000 iterations. Following Castro-Luna et al. (2007b), and Avila-Cabadilla (2011), we considered as detectors all taxa with an IV_max _≥ 0.5 and considered as indicators those taxa in which IV_max_ was statistically significant. These analyses were carried out in the R package labdsv, version 1.4-1.

#### Phyllostomids and site attributes

We examined correlations between phyllostomid response (abundance, species composition) and all the explanatory variables (season, habitat and tree basal area) using a generalized linear mixed model (GLMM). The GLMM is an extension of generalized linear models (GLMs) that include both fixed and random effects. In our models, we considered as fixed effects the season, habitat and tree basal area and as a random effect the sampling nights. In this way, we are accounting for the correlation structure caused by repeated sampling night on the same sites. For each model, we calculated Akaike's information criterion corrected for small sample size (AIC_*c*_) following Burnham and Anderson (2002). This approach allowed us to select the most plausible models from a set of models. The set of models considered for every response variable, at each scale, included the null model (without explanatory power) and other models that considered each explanatory variable independently. We compared the model using Δ_*i*_, which is the difference of AICc between a given model and the best (lowest AIC_c_) model. We also calculated the AIC weights (*w*_*i*_) for each model. The *wi* represents the weight of the evidence that a certain model is the best model given the data and the set of candidate models. The 95% confidence set of the best models was defined by summing the *w*_*i*_, from the largest to the smallest, until the sum is = 0.95. Only models with an AIC_*c*_ lower than the null model were considered to define the 95% confidence set of plausible models. All previous analyses were performed with R program (R Development Core Team 2009).

## Results

We completed 70 nights of capture effort, 34 during the dry season, and 36 during the rainy season, resulting in a total capture effort of 180 net hours in RM, RP, and P, and 140 net hours in MF habitats. Overall, 1752 individuals belonging to 28 species of Phyllostomidae were captured. The Stenodermatinae was the richest and most abundant subfamily with 16 species (57.1% of all species) and 1598 individuals (91.2% of all captures). The completeness values were above 85% for all habitats, which is considered appropriate to characterize the phyllostomid bat assemblages (Table1).

**Table 1:** Number of bats captured by species in different habitat types at Lacandona, Chiapas, Mexico. Bat guilds are as follows: F, frugivores; GI, gleaning insectivores; N, nectarivores; C, carnivores; and S, sanguivores

FAMILY Subfamily *Species*	Guild	Habitats				
		Riparian mature (RM)	Riparian pasture (RP)	Mature forest (MF)	Pasture (P)	Total
PHYLLOSTOMIDAE						
Carolliinae						
*Carollia perspicillata*	F^*s*^	20 (1.11)	34 (1.8)	5 (0.31)	1 (0.05)	60
*Carollia sowelli*	F^*s*^	9 (0.5)	6 (0.33)	8 (0.5)	2 (0.11)	25
Desmodontinae						
*Desmodus rotundus*	S	1 (0.05)	8 (0.44)	1 (0.06)	4 (0.22)	14
*Diphylla ecaudata*	S	0	1 (0.05)	1 (0.06)	0	2
Glossophaginae						
*Choeroniscus godmani*	N^*s*^	2 (0.11)	0	1 (0.06)	0	3
*Glossophaga soricina*	N^*s*^	6 (0.33)	80 (4.44)	2 (0.12)	20 (1.11)	108
*Hylonycteris underwoodi*	N^*s*^	0	0	4 (0.25)	0	4
*Lichonycteris obscura*	N^*s*^	1 (0.07)	0	0	0	1
Phyllostominae						
*Lampronycteris brachyotis*	GI^*s*^	1 (0.05)	0	0	0	1
*Lonchorhina aurita*	GI^*s*^	2 (0.11)	0	0	0	2
*Mimon crenulatum*	GI^*n*^	0	7 (0.38)	0	0	7
*Phyllostomus discolor*	GI^*s*^	0	1 (0.05)	0	0	1
*Trachops cirrhosus*	C^*s*^	2 (0.11)	0	1 (0.06)	0	3
*Tonatia saurophila*	GI^*s*^	3 (0.16)	2 (0.11)	3 (0.18)	0	8
Stenodermatinae						
*Artibeus jamaicensis*	F^*n*^	72 (4)	59 (3.27)	41 (2.56)	29 (1.61)	201
*Artibeus lituratus*	F^*n*^	113 (6.27)	190 (10.55)	25 (1.56)	74 (4.11)	402
*Artibeus phaeotis*	F^*n*^	8 (0.44)	11 (0.61)	0	6 (0.33)	25
*Artibeus toltecus*	F^*n*^	1 (0.05)	0	2 (0.12)	0	3
*Artibeus watsoni*	F^*n*^	3 (0.16)	8 (0.44)	4 (0.25)	1 (0.05)	16
*Centurio senex*	F^*n*^	1 (0.05)	1 (0.05)	2 (0.12)	1 (0.05)	5
*Chiroderma salvini*	F^*n*^	2 (0.11)	3 (0.16)	2 (0.12)	1 (0.05)	8
*Chiroderma villosum*	F^*n*^	0	3 (0.16)	1 (0.06)	0	4
*Platyrrhinus helleri*	F^*n*^	24 (1.33)	47 (2.61)	2 (0.12)	9 (0.5)	82
*Sturnira lilium*	F^*s*^	68 (3.77)	315 (17.5)	27 (1.68)	119 (6.61)	529
*Sturnira ludovici*	F^*s*^	1 (0.05)	1 (0.05)	0	0	2
*Uroderma bilobatum*	F^*n*^	48 (2.66)	92 (5.11)	0	29 (1.61)	169
*Vampyresa thyone*	F^*n*^	7 (0.38)	5 (0.27)	1 (0.06)	3 (0.16)	16
*Vampyrodes caraccioli*	F^*n*^	39 (2.16)	11 (0.611)	1 (0.06)	0	51
Total abundance		434	885	134	299	1752
Samples		18	18	16	18	70
Richness		23	21	20	14	28
Completeness(%)^1^		87	92	92	89	

Parentheses indicate the relative abundance (bats captured per night sampling) from 18 nights of sampling for RM, RP, and P, and 16 nights of sampling for MF.

Feeding strategy based on Soriano (2000); ^*n*^=Nomadic and ^*s*^=Sedentary.

^1^Based on the average of the following indices: ICE, Chao2 and Bootstrap.

**Figure 2:**
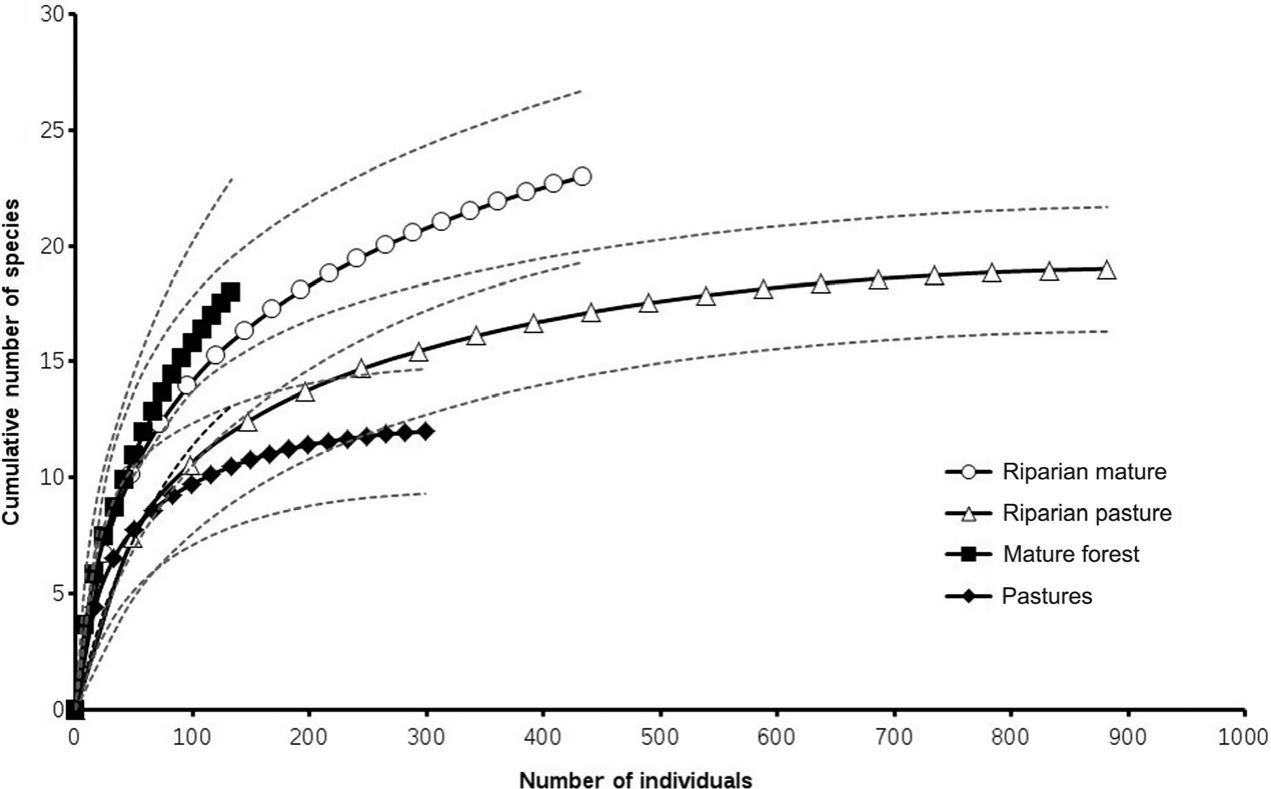
Individual-based rarefaction curves of bat species within different habitat types at the Lacandona forest. Dotted lines delineate 95% of confidence intervals.

The individual-based rarefaction curves (Fig.2) showed that we sampled all species occurring in RM and RP habitats. Captures in all habitats were dominated by five species: *Sturnira lilium* (30.1%), *Artibeus lituratus* (22.9%), *A. jamaicensis* (11.4%), *Uroderma bilobatum* (9.6%), and *Glossophaga soricina* (6.1%), which together represented 80.4% of all captures (Fig.2). Bat composition did not show a significant spatial correlation according to the Mantel test (*R* = 0.057, *P* = 0.27).

### Bat assemblages and guilds

**Figure 3:**
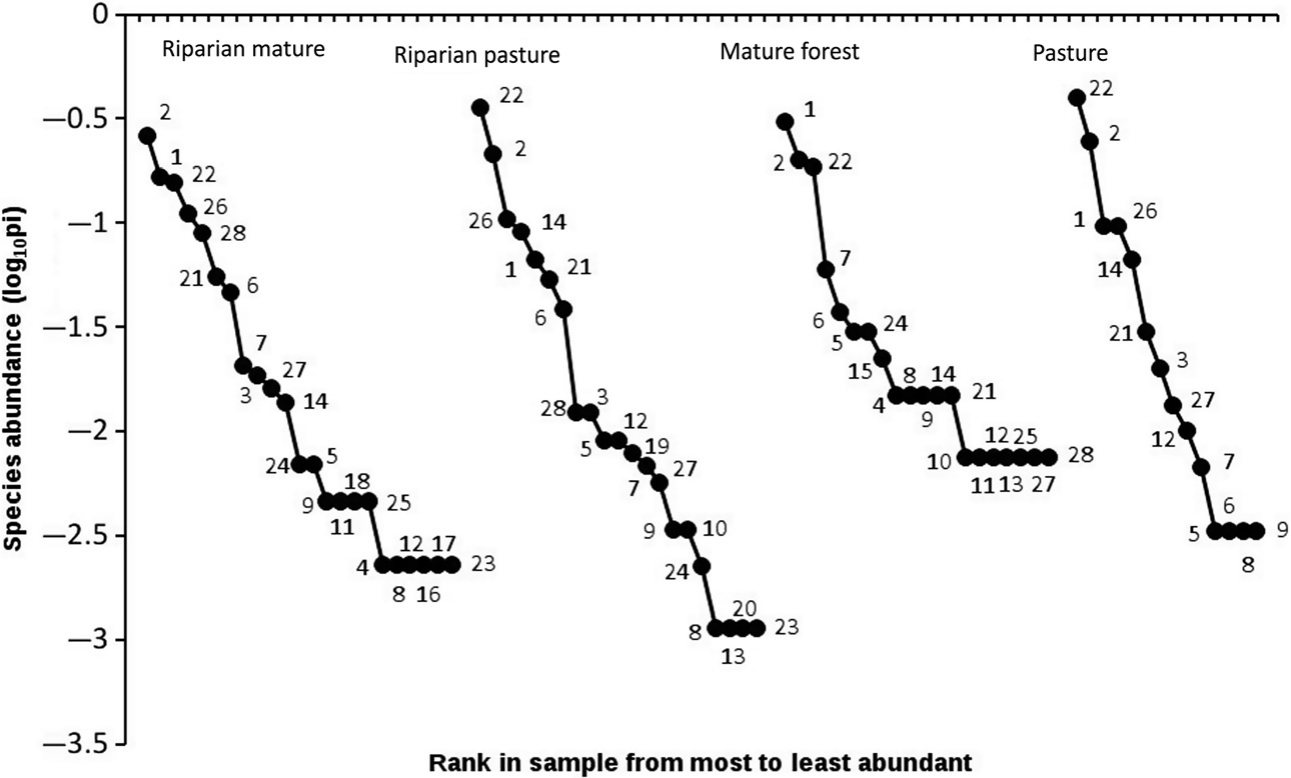
Rank-abundance (dominance-diversity) curves of bat species at Lacandona forest. Numbers represent the species captured. 1: *Artibeus jamaicensis*, 2: *A. lituratus*, 3: *A. phaeotis*, 4: *A. toltecus*, 5: *A. watsoni*, 6: *Carollia perspicillata*, 7: *C. sowelli*, 8: *Centurio senex*, 9: *Chiroderma salvini*, 10: *C. villosum*, 11: *Choeroniscus godmani*, 12: *Desmodus rotundus*, 13: *Diphylla ecaudata*, 14: *Glossophaga soricina*, 15: *Hylonicteris underwoodi*, 16: *Lampronycteris brachyotis*, 17: *Lichonycteris obscura*, 18: *Lonchorhina aurita*, 19: *Mimon crenulatum*, 20: *Phyllostomus discolor*, 21: *Platyrrhinus helleri*, 22: *Sturnira lilium*, 23*: S. ludovici*, 24: *Tonatia saurophila*, 25: *Trachops cirrhosus*, 26: *Uroderma bilobatum*, 27: *Vampyressa thyone*, and 28: *Vampyrodes caraccioli*.

Species richness declined from 23 species in RM, to 21 in RP, to 20 in MF and 14 species in P, resulting in a total of 28 species (Fig.3). Habitats shared 10 species. The RM habitat presented three exclusive species; in addition, the RP had two exclusive species and the MF one exclusive species, while open pastures had none. Species of the subfamily Phyllostominae were absent from open pastures (Table1).

**Figure 4:**
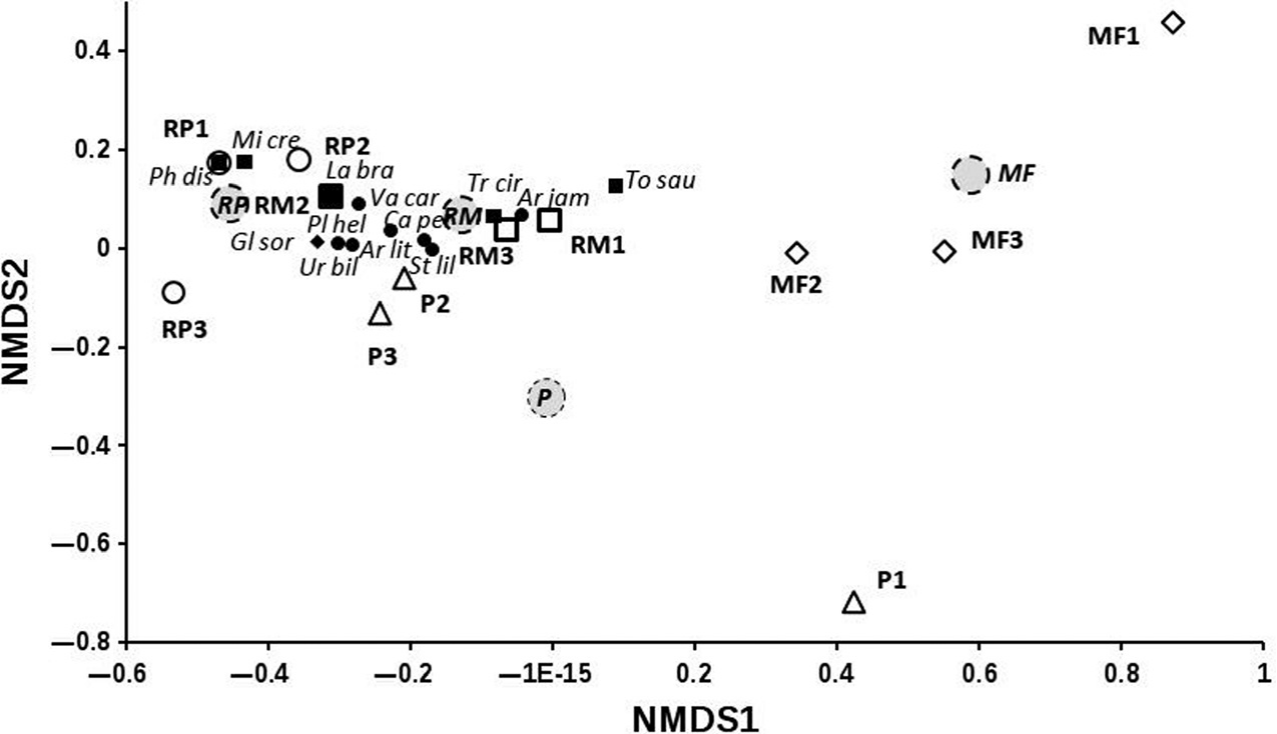
NMDS ordination based on species composition and bat abundance data at the Lacandona forest, where RM, riparian mature; RP, riparian pasture; MF, mature forest; P, open pasture.

Frugivores accounted for the greatest percentage of both captured species and individuals (59.2% of species and 90.3% of individuals), followed by gleaning insectivores (18.5% of species and 0.7% of individuals) and nectarivores (11.1% of species and 7.7% of individuals). Sanguivores were represented by *Desmodus rotundus* with 11 individuals and *Diphylla ecaudata* with two individuals; in addition, carnivores were represented only by *Trachos cirrhosus* (Table1). The number of bat guilds per habitat type declined from five in the RM and the MF, to four in RP, and three guilds in P (Fig.4).

### Bats as indicator taxa

Six of the 18 species recorded could be considered as habitat indicators (Table2). The subfamilies Glossophaginae and Stenodermatinae were tightly associated with RP; however, at the genus level *Glossophaga* was mostly associated with MF, and *Sturnira* was tightly associated with RP.

**Table 2:** Bat taxa with a significant indicator value (IV) in the Lacandona, Chiapas, Mexico

Taxonomic level	Taxon	Habitat	IV	*P*-value	Category
Subfamily	Glossophaginae	RP	0.682	**0.048**	I
	Stenodermatinae	RP	0.500	**0.025**	I
	Desmodontinae	RP	0.548	0.271	D
Genus	Glossophaga	MF	0.739	**0.038**	I
	Lonchorhina	RM	0.666	0.192	D
	Mimon	RP	0.666	0.170	D
	Platyrrhinus	RP	0.573	0.321	D
	Sturnira	RP	0.593	**0.016**	I
	Uroderma	RP	0.544	0.087	D
	Vampyrodes	RM	0.761	0.212	D
Species	*C. perspicillata*	RP	0.559	0.215	D
	*G. soricina*	RP	0.739	**0.036**	I
	*L. aurita*	RM	0.666	0.186	D
	*M. crenulatum*	RP	0.666	0.183	D
	*P. helleri*	RP	0.573	0.337	D
	*S. lilium*	RP	0.593	**0.019**	I
	*U. bilobatum*	RP	0.544	0.089	D
	*V. caraccioli*	RM	0.761	0.222	D

RM, riparian mature; RP, riparian pasture; MF, mature forest; P, open pasture; D, detector taxon; I, indicator taxon.

Significant *P*-values (<0.05) appear in bold.

We identified twice as many detector taxa as indicator taxa (Table2). The subfamily Desmodontinae was associated with RP. We found *Lonchorhina* and *Vampyrodes* were associated with RM, and *Mimon*, *Platyrrhinus* and *Uroderma* were associated with RP. Finally, most of the detector species were associated with RP (*Carollia perspicillata*, *Mimon crenulatum*, *Platyrrhinus helleri,* and *U. bilobatum*), and only two species were associated with RM (*Lonchorhina aurita* and *Vampyrodes caraccioli*).

### Response to habitat attributes

Three axes were considered in the NMDS ordination (Fig.4, stress = 2.3). We only used scores from axes 1 and 2 for the construction of the biplot. Axis 1 of the plot separated riparian and nonriparian habitats. Riparian pasture and RM habitat are closer together, showing more similarity in species composition than MF and P, which are strongly separated (Fig.4).

The assemblage dissimilarities represented by NMDS (Fig.4) were significantly associated with the type of habitat and total basal area of the vegetation. Phyllostominae species such as *M. crenulatum*, *P. discolor*, *Lampronycteris brachyotis*, and *T. saurophila* were associated with the sites with the greater basal area (Table3). Sites with high basal area were the riparian habitats: RM with 25639.51 m^2^/0.1 ha and RP with 20,285.77 m^2^/0.1 ha. On the other hand, whereas in nonriparian habitats basal area was 8561.03 m^2^/0.1 ha in P and 6241.88 m^2^/0.1 ha in RM. Also, the variation in bat abundance was positively associated with the rainy season (Table3).

**Table 3:** Confidence set of plausible models (95%) explaining the variation in response variables

Response variable	Model	*K*	logLik	AIC_*c*_	Δ_*i*_	*w*_*i*_
Night data analyses						
Abundance	Season	3	450.82	910.64	0.00	0.81
	Habitat + Season	6	442.39	913.58	2.93	0.19
	Habitat	5	500.12	1020.24	109.59	0.00
Site data analyses						
Species composition	Habitat	5	2.12	15.75	0.00	0.56
	*V*_basal area_	3	4.35	17.70	1.94	0.21

*K*, number of estimated parameters; logLik, log-likelihood; AIC_*c*_, sample-sized adjusted Akaike information criterion; Δ_*i*_, Akaike differences; and *w*_*i*_, Akaike weights. Response variables: species abundances, species rarified at lowest number of captures, species composition, and guild composition. Explanatory variables are the following: habitat, *V*_spcomp_, using scores of NMDS axis 1; *V*_height_, average height of trees at each site; *V*_basal area_, total basal area at each site; *V*_abundance_, total number of trees; *V*_rich_, number or species.

## Discussion

Our results suggest differences in species richness and abundance of phyllostomid bats between riparian and nonriparian habitats. This suggests that riparian corridors in agricultural landscapes allow the persistence of sensitive phyllostomines, which demonstrates the importance of maintaining different vegetation cover types to conserve bat biodiversity in areas under agricultural land use (Medina et al. 2007; Williams-Guillén and Perfecto 2010).

### Bat assemblages

There was a clear association of riparian habitats with greater richness and abundance of bat species (Seaman and Schulze 2010). The high species richness and number of individuals recorded in a riparian habitat corroborates their significance as flyways corridors within fragmented landscapes (Limpens and Kapteyn 1991). Riparian corridors offer a great diversity of chiropterophilic and chiropterochoric resources (Sánchez-Merlo et al. 2005) and provide roosting sites, commuting habitats, water, and refuge from adverse climatic conditions and predators (Estrada and Coates-Estrada 2002; Galindo-González and Sosa 2003).

We found more nomadic bats (Stenodermatinae) in anthropogenic habitats as they frequently move among forest remnants probably searching for food, which increases their capture probability (Table1 Soriano (2000)). This suggests that Stenodermatinae but especially *Artibeus* and *Sturnira* may facilitate forest regeneration in open pastures through seed dispersal (García-Morales et al. 2012). Nonetheless, *V. caraccioli* (Stenodermatinae) was caught exclusively in undisturbed forest, and *C. perspicillata* (Carolliinae) was caught mainly in riparian pasture habitat, supporting the idea that sedentary bats seldom leave mature forests (Soriano 2000). In this context, open pastures generate impermeability, because they seem to limit the movement of frugivorous bats restraining seed-flux between fragments and their matrix process that is crucial in the recovery in old fields (Cortés-Delgado and Pérez-Torres 2011).

The most abundant species in pastures were *A. lituratus*, *C. perspicillata,* and *S. lilium*. These species are common and may be less susceptible to habitat disturbance because of their generalist diet, and their probability of arriving to modified areas is higher compared to rare species. Species such as *Uroderma bilobatum* was associated with all habitat types reflecting its capacity to consume fruits from primary and secondary forests (Gorresen and Willig 2004).

### Habitat disturbance and bat guilds

Frugivores were the best represented trophic guild in all habitats. The feeding habits of the Phyllostomid frugivorous species make them highly tolerant to human-disturbed habitats, and they can easily recolonize disturbed areas (Avila-Cabadilla et al. 2009). We found a higher abundance of some frugivorous species (*A. lituratus*, *P. hellery*, *S. lilium,* and *U. bilobatum*) in RP than in the other habitats. In the case of *A. lituratus*, the guild of large fig-eating bats (genus *Artibeus*) showed greater tolerance to fragmentation than other frugivorous phyllostomids, because of its foraging strategy and capacity for flying long distances (Cosson et al. 1999; Avila-Cabadilla et al. 2012). In contrast, gleaning insectivores and carnivores (Phyllostominae) preferred mature forest instead of human-disturbed habitats (García-Morales et al. 2013). In our study, gleaning insectivores and carnivores were absent from open pastures, perhaps as a consequence of food scarcity, shelter, and roost resources (Medellín et al.^2000^; Schulze et al. 2000; Gorresen and Willig 2004; de la Peña-Cuéllar et al. 2012). Gleaning insectivores were present, however, in RP, supporting our hypothesis that these habitats offer good sites for lurking and hunting for prey (Gorresen and Willig 2004; García-Morales et al. 2013).

### Bats as indicator taxa

Taxonomic level analysis demonstrates that in the study area phyllostomid bats are poor ecological indicators (Castro-Luna et al. 2007b). Contrary to other findings (Fenton et al.^1992^; Medellín et al. 2000) that suggest the Phyllostominae subfamily is an ecological indicator of habitat modification because of strong association with preserved forest, our results suggest that generalist abundant species like the subfamilies Glossopaginae and Stenodermatinae may be better as ecological indicators. Frugivorous bats like Stenodermatinae, which can fly over large distances and visit different vegetation types (Estrada and Coates-Estrada 2002), could be useful detector taxa for studies aimed at evaluating different degrees of disturbance, rather than highly specialized taxa in which populations decline rapidly under environmental changes (Mcgeoch et al. 2002; Castro-Luna et al. 2007b).

Nectarivorous *G. soricina* and frugivorous *S. lilium* can be both considered as indicator taxa of habitat change in RP. These species can forage in areas with a simple vegetation structure but with high abundance of chiropterophilic and chiropterocoric species, in particular, *S. lilium* that usually feeds on understory shrubs and pioneer tree species (Marinho-Filho 1991).

### Habitat attributes

Despite the relatively stable climatic conditions throughout the year in tropical rain forests, the availability of resources varies seasonally, and our results suggest that bats could be forced to make adjustments in their foraging strategy to cope with seasonal variations of resources availability such as food and roosts (Ramos-Pereira et al. 2010). Frugivores diet varies over the year and throughout their geographic ranges as the abundance and availability of fruit species change (Bonaccorso 1979). Seasonal variation could be a cause of variation in resource abundance and diversity, reproductive constraints and forest fragmentation, intensifying the severity of seasonal changes in source availability, furthermore, this variation could be a cause of shifts in foraging strategy that may not be needed in unfragmented landscapes (Klingbeil and Willig 2010).

The positive relationship between species composition and basal area of trees can be explained by the preference of some bat species for roosting in large trees. Large trees provide more potential roost sites because there is a close relationship between tree size and the number of natural cavities (Evelyn and Stiles 2003; Ortiz-Ramírez et al. 2006).

### Conservation implications

Our results suggest that more efforts are needed to preserve riparian corridors in order to conserve Neotropical bats in human-dominated landscapes. Specifically, increasing matrix heterogeneity at larger spatial scales through the retention and protection of riparian forests is important (Akasaka et al. 2012). In human-dominated landscapes, riparian vegetation is highly threatened by agricultural practices, cattle concentration, extraction of firewood and timber. This suggests that conservation strategies should provide incentives for landowners to conserve and restore riparian vegetation in their properties (Harvey et al. 2006). Maintaining diverse populations of bats in human-dominated landscapes can benefit agricultural practices via seed dispersal and pollination services and by limiting arthropod populations (Perfecto and Vandermeer 2008; Williams-Guillén et al. 2008). Additionally, management programs in human-dominated landscapes should also focus on the quality of the remaining riparian vegetation as a suitable breeding habitat for bats and other animals (Bolívar-Cimé et al. 2013).
